# A multi‐technique study of corrosion products at the steel–concrete interface under two exposure conditions

**DOI:** 10.1111/jmi.13100

**Published:** 2022-03-28

**Authors:** Zhidong Zhang, Patrick Studer, Ueli Angst

**Affiliations:** ^1^ Institute for Building Materials ETH Zurich Zurich Switzerland; ^2^ Institute of Structural Engineering ETH Zurich Zurich Switzerland

**Keywords:** aggregate effect, concrete, corrosion products, durability, iron hydroxides, oxygen depletion

## Abstract

Steel corrosion can cause serious damage to reinforced concrete structures. This study employed multiple techniques, including SEM/BSE, EDX and Raman spectroscopy, to analyse the distribution and mineral composition of corrosion products (rusts) in corroded reinforced cementitious materials under two conditions, namely, chloride‐induced corrosion and accelerated corrosion in carbonated mortar. Results showed that corrosion products tend to precipitate in large pore spaces close to the steel bar, such as the bleed water zones and voids. Corrosion products initially grew on the walls of these large pores and then the interior was filled with needle‐like products gradually. In carbonated mortar, the length of some corrosion layers matches well the size of the coarse aggregate close to the steel. The main phases that were identified based on Roman spectra are magnetite and maghemite (after samples were exposed to atmosphere). Siderite was observed in carbonated mortars, which is not commonly found under natural conditions.

## 1 INTRODUCTION

Corrosion induced damage is one of the main deterioration mechanisms of reinforced concrete structures. After the initiation of corrosion, the formed corrosion products (rusts) may lead to expansive stresses and eventually crack the concrete cover. Traditionally, the expansive stresses models assume that corrosion products only grow at the steel surface.^1^ However, this assumption is not appropriate because the process of corrosion involves soluble species that can dissolve in the concrete pore solution and migrate or diffuse through the concrete away from the corroding steel.^2^, ^3^ In agreement with this, images from scanning electron microscopy (SEM) revealed that precipitated corrosion products were found at distances of up to the mm range from steel and even farther away in cracked concrete.^4^, ^5^ Therefore, it is important to observe the distribution of corrosion products, to analyse the affecting factors, and to identify species of corrosion products. The previous studies generally used SEM equipped with a backscatter detector (BSE) and energy dispersive X‐ray analysis (EDX) to study the distribution of corrosion products.^4^, ^5^ To further identify the chemical composition, this study tried multiple techniques, including SEM/BSE, EDX and Raman spectroscopy, to analyse corrosion products formed at the steel–concrete interface in corroded reinforced cementitious materials under two conditions, namely, chloride‐induced corrosion and accelerated corrosion in carbonated mortar.

## 2 EXPERIMENTS

Two types of specimens were studied. Concrete specimens with carbon steel bars were retrieved by core drilling from a concrete bridge deck in the Swiss Alps, with age of more than 40 years.^6^ The concrete was made of CEM I cement and water‐to‐cement ratio (*w*/*c*) of 0.5. The cylindrical specimens were immersed in the NaCl solution (3.5 wt.%) until the initiation of corrosion.^6^

**FIGURE 1:**
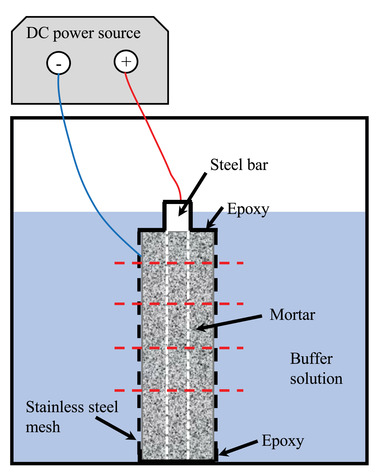
Experimental setup of accelerated corrosion for carbonated mortar. The dashed red horizontal lines indicate the cutting locations. The ends of the steel were protected by epoxy resin to avoid the direct contact with liquid

Mortar specimens were prepared in the laboratory with CEM I cement, *w*/*c* = 0.5, and sand‐to‐cement ratio of 2. After casting, specimens were stored in a climate room with the relative humidity of 95% for 1 year. The cylindrical specimens, ∼ 1 cm in diameter with an embedded 0.3 cm carbon steel bar (sample preparation followed the procedure in the previous study^7^), were then carbonated in a chamber with 4% CO_2_ and 58% relative humidity. After carbonation, cylinders were immersed in a buffer solution (0.1 M Na(HCO_3_)_2_ + 0.01 M NaCO_3_) with pH ∼ 9.0 in a cup for accelerated corrosion experiment with an impressed current density of 100 μA/cm^2^ for 12 days (see Figure 1 for the setup). After accelerated corrosion, cylinders were then immediately cut with 0.5 cm interval from top (indicated in Figure 1 with red lines) and quickly prepared for the following tests.

Because of different hardness and wear rates between steel and concrete, the sample preparation for SEM/BSE, EDX and Raman spectroscopy measurements followed a well‐designed procedure for polishing and grinding, which can be found in the previous study.^7^ A FEI Quanta 600 environmental SEM with a BSE detector was used to image the polished surface. After SEM imaging, the selected spots were scanned by Raman spectroscopy (Horiba: LabRAM HR Evolution UV‐VIS‐NIR) with an acquisition time of 60 s and wave number from 150 to 1200 cm^–1^. The measured Raman spectrum was compared with available spectra in the literature^8^ so that the mineral composition could be identified.

## 3 RESULTS

### 3.1 Chloride‐induced corrosion

**FIGURE 2:**
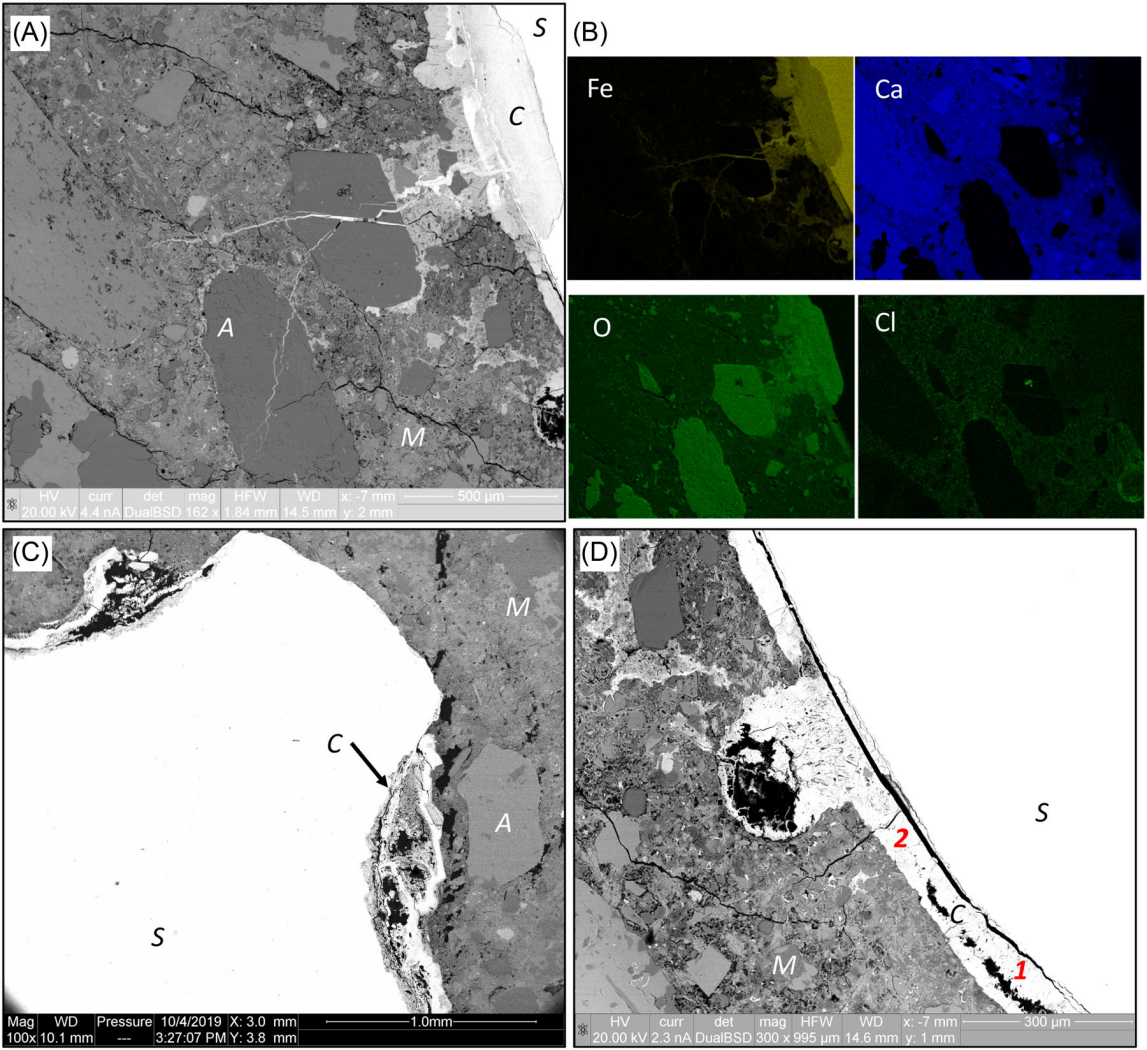
SEM/BSE and EDX results of chloride‐induced corrosion: (A) the effect of aggregates and cracks on corrosion products distribution (S = steel, C = corrosion layer, A = aggregate, and M = mortar); (B) EDX results of elements mapping in A; (C) corrosion products distribution at a rib and (D) corrosion products in a void and the bleed water zone

With the help of EDX pointing and mapping techniques, we were able to identify the corrosion products in SEM/BSE images. In the EDX results, corrosion products occupied regions that displayed high content of Fe and O. These areas show an intermediate brightness in the SEM/BSE images (see an example in Figure 2A and B). For the concrete specimens, the corrosion products non‐uniformly distribute around the rebar. Significant amounts of corrosion products were observed in a few locations. A spot is near the ribs of the steel as shown in Figure 2C. The gaps around the steel rebar, such as the bleed water zones, are either partly or completely filled with corrosion products (see Figure 2D). If a void is located near the steel surface or even touching the steel surface, a noticeable amount of corrosion products could be found in it. These zones and voids were initially likely filled with pore solution during the accelerated corrosion tests. Without sufficient protection by solids, steel at these region is more easily corroded than the low porosity regions.^9^ Furthermore, cracks through the cement paste also have an impact on the distribution of corrosion products (see Figure 2A). Cracks starting from the steel surface sometimes affect corrosion products distribution. Through these channels, the corrosion products can move long distances away from the steel.

**FIGURE 3:**
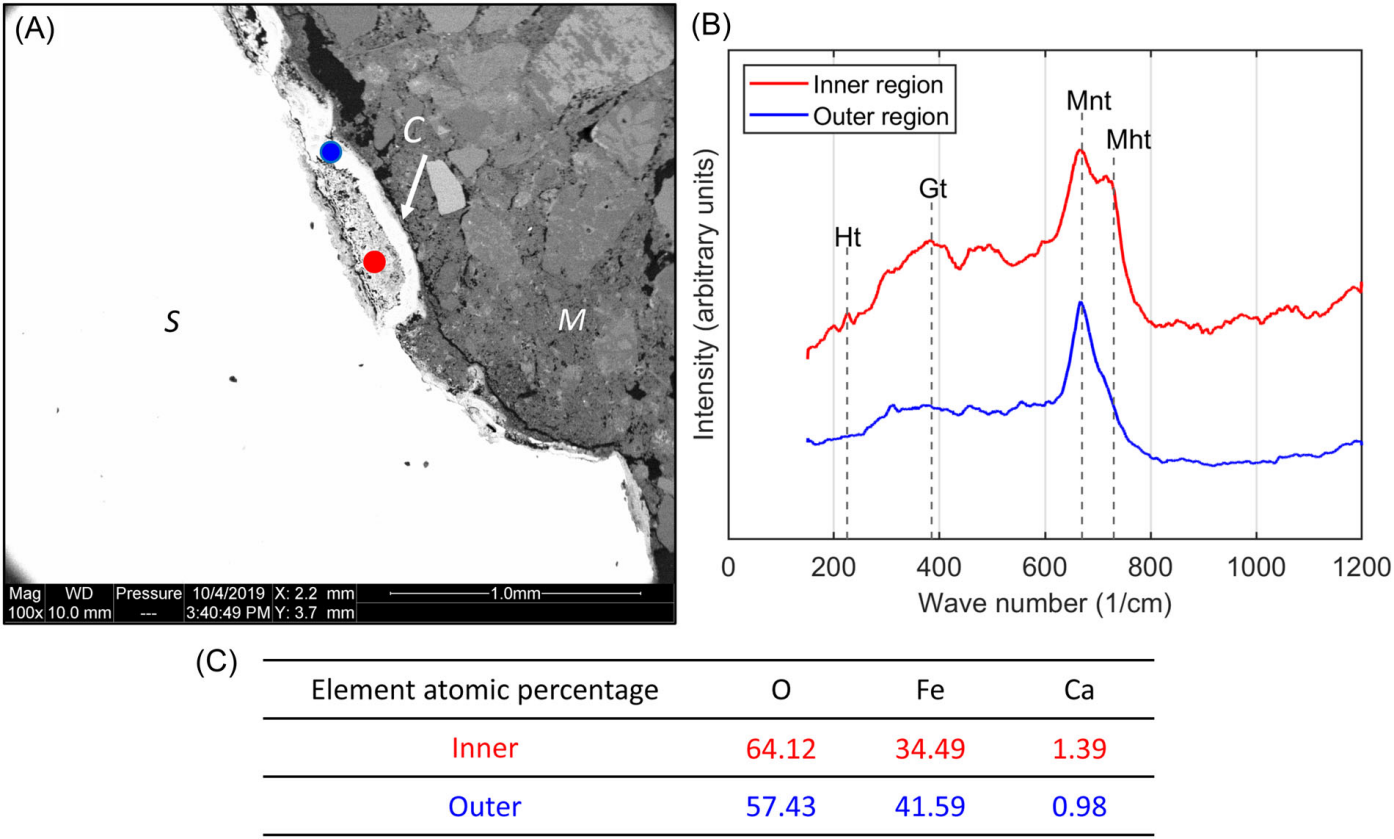
Raman and EDX results for chloride‐induced corrosion: (A) corrosion layer on steel surface with two locations for Raman spectroscopy measurements, (B) measured Raman spectra (Ht = hematite, Gt = goethite, Mnt = magnetite, and Mht = maghemite) and (C) EDX results of element atomic percentage for inner and outer regions

Morphology of the corrosion products in the large gaps and voids displays an visible outer dense ring (much brighter regions in Figure 2C and D), while in‐between there are some more porous materials (less bright regions), with the needle‐like corrosion products inside (see the region labelled with 1 in Figure 2D). The needles seem to grow from the dense ring towards the interior of the void. With ongoing corrosion, the growth of the needle‐like products tend to close the gap between the steel and the outer dense ring and eventually these areas are filled (see the region labelled with 2 in Figure 2D and the blue point in Figure 3A). The thickness of the corrosion layer was here about 100 μm, but it should be noted that this depends on the available space for the growth of corrosion products, that is, the size of the bleed water zone and the air void. The more the steel is corroded, the more space becomes available. The growth of corrosion products in the voids follows the same procedure. Within the SEM/BSE images, it is clearly visible that before filling the whole space, the products tend to grow along the walls of the macroscopic voids.

These gaps between steel and mortar eventually fill with the corrosion layer which mainly contains Fe and O as confirmed by EDX elements mapping in Figure 2B. Some corrosion products can penetrate into the mortar matrix, in which, iron, oxygen and calcium were detected by EDX mapping as shown in Figure 2B. Even though these specimens were in the very early stage of corrosion initiation, the transport of corrosion products through mortar is visible. With the increase of corrosion time, more corrosion products can transport to farther away from the steel surface.

The main goal of Raman spectroscopy was to identify the mineralogical phases within the corrosion products. The distribution of corrosion products in SEM/BSE images in Figure 2 clearly shows two different regions, outer dense ring (much brighter) and inner porous region (less bright). Therefore, spot analyses of handpicked locations were done on the outer (blue dot) and inner (red dot) regions, as indicated in Figure 3A. Their Raman spectra in Figure 3B show that both have a main peak at 665–670 cm^–1^, while the inner products show an extra main peak at 730 cm^–1^ and small peaks at 225 and 385 cm^–1^. Comparing to the reference spectra,^8^ the main characteristic peaks are identified as magnetite (Fe_3_O_4_, at 670 cm^–1^) and/or maghemite (γ‐Fe_2_O_3_, at 665 and 730 cm^–1^) and the small peaks are from hematite (Fe_2_O_3_), goethite (α‐FeO(OH)), and maghemite, respectively. EDX results of element atomic percentage in Figure 3C show that the Fe/O ratio of the outer region is very close to that in magnetite, while the inner region has much higher content of oxygen. Therefore, it is concluded that the outer ring mainly consists of magnetite, while the inner product is a mixture of magnetite and/or maghemite, maybe with a small amounts of hematite and goethite. The phase differences in the distribution of corrosion products may be related to the growth stage of corrosion products as mentioned above. However, only based on results in this study, a definitive interpretation is difficult to be provided.

### 3.2 Impressed current corrosion in carbonated mortar

**FIGURE 4:**
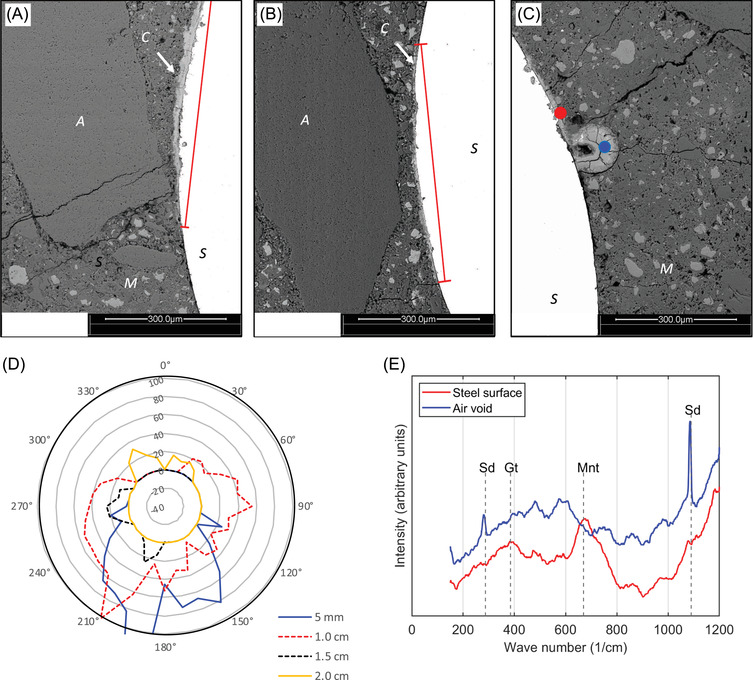
Results of impressed current corrosion in carbonated mortars: (A), (B) The effects of aggregates on corrosion products distribution (red lines indicate the length of corrosion layer); (C) corrosion products distribution in a void and at steel surface (indicating locations for Raman spectroscopy measurements); (D) thickness of corrosion layer at different depth from the top and (E) measured Raman spectra (Sd = Siderite)

Similar to Figure 2, the corrosion layer around the steel bar is very non‐uniform, as seen in Figure 4. The effect of coarse aggregates on the distribution of corrosion products is clearly shown in Figure 4A and B. The length of the corrosion layer exactly follows the shape of the coarse aggregate. The end of the corrosion layer matches very well with the end of the aggregate. If there is a void present near the steel surface, corrosion products are formed in the void (see Figure 4C).

The thickness of the corrosion layer around the steel bar can be measured at different angles. With a spacing of 10°, the corrosion layer was measured with an accuracy of 2 μm for each cutting surface. As starting point, the location where the steel bar has the thickest mortar cover was chosen as 0°, meaning that the opposite side has the thinnest cover (180°). Therefore, it is possible to compare the thickness of corrosion layer for all slices at different depths from the top of the specimen. As shown in Figure 4D, the thickness of corrosion layer varies a lot with depth and angle. The general trend is that the overall thickness decreases with the depth of slice. For instance, the slices at depth of 0.5 and 1.0 cm have much thicker corrosion layers than the two following ones. Figure 4B also shows that more corrosion products were in the region with the thinnest concrete cover (around 180°). This behaviour is obvious in slices 1, 2 and 3, where no corrosion occurs on the side with the largest concrete cover. These results show a correlation between the cover thickness and the presence of corrosion products, which may be explained by the fact that the region with the thickest cover has a comparatively high electrical resistance. Therefore, in the accelerated corrosion test, more polarisation current can pass through the regions with thinner cover and lead to more pronounced corrosion in these zones.

Corrosion products in a void and at the steel surface as indicated by blue and red dots in Figure 4C were analysed by Raman spectroscopy. Products in the void have four obvious peaks as shown in Figure 4E. Peaks at 287 and 1090 cm^–1^ correspond to siderite (FeCO_3_), while 481 and 574 cm^–1^ are not the characteristic peaks of any corrosion products. Therefore, the main phase precipitated in the void is siderite, formed in a carbonate ion‐rich solution.^10^ The products close to the steel surface show a clear peak at 670 cm^–1^ and a less obvious peak at 385 cm^–1^, which are the characteristic peaks of magnetite and goethite,^8^ so this corrosion layer may be a mixture of these two minerals. It should be noticed that most peaks in Raman spectra are very broad (except siderite), indicating that these phases have a low crystallinity.^11^

## 4 DISCUSSIONS

### 4.1 Effects of aggregates on corrosion products distribution

An interesting finding is that more corrosion products can be found on steel surface where a coarse aggregate is close, in particular for the impressed current corrosion in carbonated mortars. Effects of coarse aggregates on the chloride‐induced steel corrosion have been studied by Razmjoo and Poursaee,^12^ which reported that decreasing the distance between aggregates and steel had adverse impact on the corrosion initiation on the steel. They claimed that the presence of steel bars acted as a barrier to chlorides transport and chlorides are trapped at the steel surface, leading to a more rapid increase in chlorides concentration between the coarse aggregate and the steel bar. However, the EDX mapping results of Cl in Figure 2B, showing less Cl between the coarse aggregate and steel than the bulk mortar, does not support this explanation. For the carbonated mortars, there were no chlorides but they still shows the effect of coarse aggregates. After Fe^2+^ ions are forced to leave the steel, they diffuse into the mortar matrix or precipitate as ferrous hydroxides (Fe(OH)_2_) if the concentration of ferrous ions (Fe^2+^) exceeds a certain level.^3^, ^13^ The aggregate hinders Fe^2+^ diffusion and thus corrosion products can only precipitate between the aggregate and steel. It may be also caused by the oxidation of Fe^2+^ that still consumes a certain amount of oxygen even though the cathodic reaction (oxygen reduction) is not needed in the impressed current experiments. With the decrease of oxygen concentration, more Fe^2+^ ions can diffuse into the mortar matrix. This further reduces the concentration of Fe^2+^ near the steel surface, which may favour the release of Fe^2+^ from the steel if there is a coarse aggregate nearby. Therefore, we believe the local corrosion rate may vary with ions concentration or steel properties.

### 4.2 Mineral composition of corrosion products

The observed corrosion products may be different from these in the natural corrosion as oxygen is always playing an important role during sample preparation. Corrosion is an electrochemistry process, always starting with the release of Fe^2+^ from the steel due to the loss of the passive layer. Then, Fe^2+^ can directly precipitate as Fe(OH)_2_ in concrete. If oxygen is not enough, the Schikorr reaction may happen, in which Fe(OH)_2_ is oxidised to form magnetite by protons of water.^14^ If oxygen is available, Fe^2+^ is oxidised to form ferric oxyhydroxide phases, such as goethite (α‐FeO(OH)), which is further either topotactically transformed into hematite and maghemite through dehydration^15^ or reduced to magnetite if enough Fe^2+^ is still present in the solution.^16^ Meanwhile, the partial oxidation of Fe(OH)_2_ suspension leads to the precipitation of magnetite as well.^17^ Therefore, depending on the reaction stage and the availability of oxygen, we may see different corrosion products.

For the accelerated corrosion in the carbonated mortar, siderite was observed, which can only form under anoxic conditions due to the need for reduced, dissolved Fe^2+^ reacting with the dissolved inorganic carbon (e.g. CO_3_^2−^).^10^ In addition to a source of dissolved iron, the favourable pH to form siderite is slightly above 7.2,^18^ which is in the pH range of the buffer solution and the pore solution in the carbonated mortar. Generally, the observed siderite is not a pure phase and often contains a mixture of carbonate mineral phases, such as calcium carbonate.^18^ From this point, corrosion products in the void (see the blue dot in Figure 4C) could be mixed with calcium carbonate as the Ca‐rich siderite has been reported in loosely precipitated corrosion layer.^19^ In reinforced concrete, siderite is an intermediate corrosion product, which is eventually oxidised to magnetite and/or goethite.^20^

## 5 CONCLUSION

In this study, the distribution and mineral composition of corrosion products in two experimental regimes, namely, chloride‐induced corrosion and accelerated corrosion in carbonated mortar, were analysed by SEM/BSE, EDX and Raman spectroscopy. We conclude:

1. A clear corrosion layer can be seen in some regions at the steel surface. The distribution of corrosion products is very non‐uniform, which may depend on various factors, such as ion concentration, local steel properties, the microstructure of mortar close to the rebar, the availability of oxygen, etc.
2. Corrosion products tend to precipitate in empty spaces at the steel surface, such as bleed water zones and voids. Corrosion products initially grew on the walls of these regions and then the interior is filled with needle‐like products gradually.
3. If a coarse aggregate is close to the steel, more corrosion products can be found in the zone between the aggregate and the steel, in particular for the impressed current corrosion in carbonated mortar. This may be related to ion and oxygen diffusion.
4. The main phases in both experimental regimes are magnetite and maghemite. Siderite was also identified in carbonated mortars.
